# Feature Review on Food Nutrition: Innovative Perspectives on Bioactive Compounds, Consumer Health, and Food Sustainability

**DOI:** 10.3390/foods15122114

**Published:** 2026-06-11

**Authors:** Nieves Baenas, Diego A. Moreno

**Affiliations:** 1 Department of Analytical Chemistry, Nutrition and Food Sciences, University of Alicante, San Vicente del Raspeig, 03690 Alicante, Spain; nieves.baenas@ua.es; 2 Phytochemistry and Healthy Food Lab (LabFAS), Food Science and Technology Department, CEBAS-CSIC, Campus Universitario de Espinardo-25, 30100 Murcia, Spain

The field of food science and nutrition is currently experiencing a profound transformation driven by the growing demand for healthier, safer, more sustainable, and consumer-oriented foods. Recent developments in food and nutritional sciences increasingly rely on foodomics and other advanced analytical approaches to improve food quality and safety, identify adulteration and fraud, and deepen the understanding of how bioactive food compounds influence human physiology at the molecular level. Simultaneously, climate change and environmental sustainability are placing significant pressure on global food supply chains, particularly in vulnerable regions, making it essential to explore alternative ingredient sources, promote the valorization of agro-industrial by-products, and implement sustainable agricultural and food production systems capable of ensuring safe, nutritious, and sustainable diets [1]. Advances in nutrition research, analytical chemistry, food processing technologies, and personalized health approaches are reshaping the way scientists understand the relationship between food and human health. Within this context, this Special Issue provides an innovative and forward-looking perspective of current research trends related to nutrition, bioactive compounds and metabolism, aromatic compounds and their relationship with consumer preferences, dietary strategies for the management of health conditions and diseases, as well as emerging non-thermal technologies capable of transforming food production. Collectively, these advances aim to enhance nutritional quality, processing sustainability, food safety, and overall human health.

The articles included in this Special Issue collectively highlight the multidisciplinary nature of modern food science. Current and future perspectives in food and nutrition demand highly interdisciplinary research and innovation strategies that integrate areas such as biomarkers discovery, nutrigenetics, dietary personalized guidelines, population health, and sustainable food systems. A comprehensive understanding of the relationships between dietary habits, health, and well-being across the entire life cycle requires integrated scientific approaches combining nutrition and food science, medicine, and technological innovation. Beyond traditional nutritional approaches focused solely on nutrient composition, contemporary research increasingly integrates metabolomics, microbiota interactions, sensory sciences, and toxicological assessment, to achieve a more comprehensive understanding of the impact of diet on human health. The studies presented herein demonstrate how food research is moving toward a more holistic understanding of novel dietary patterns, food innovation, and human health.

One of the most innovative perspectives explored in this Special Issue concerns the study of spices and aromatic herbs, not only as contributors to organoleptic properties but also as modulators of human emotions and physiological responses. Traditionally, herbs and spices have been investigated mainly for their sensory attributes and culinary applications. However, recent evidence suggests that aromatic compounds can also influence emotional perception and consumer acceptance in complex ways. In this regard, researchers combined headspace solid-phase microextraction (HS-SPME) and gas chromatography–mass spectrometry (GC-MS) techniques to identify volatile compounds responsible for aroma profiles, together with sensory analysis performed by trained panelists. Importantly, the investigation integrated traditional sensory methodologies with physiological and emotional assessment tools. This comprehensive approach allowed researchers to evaluate not only aroma intensity and global acceptance but also emotional and physiological responses elicited by the tested samples (contribution 1). Such an integrative vision significantly enriches research in consumer sciences and food product development. Understanding the emotional dimension of food perception may contribute to the design of personalized products, neuromarketing strategies, and improved prediction of consumer preferences. The incorporation of emotional and physiological biomarkers into sensory science represents a novel and promising research avenue that could profoundly influence future food innovation.

Despite the growing interest in the beneficial effects of spices and aromatic herbs, their dietary consumption is often associated with traditional use rather than evidence-based toxicological assessment. Many aromatic plants contain naturally occurring compounds that, although consumed in small quantities, may generate toxicological or metabolic effects under certain conditions. Therefore, ensuring food safety remains a crucial aspect of food innovation. An important contribution included in this Special Issue addresses the risk assessment of pulegone, a naturally occurring compound present in essential oils from pennyroyal mint (*Mentha pulegium*) and other aromatic plants used in herbal infusions and flavoring products. The authors analyzed available toxicological data together with estimates of dietary exposure in humans, comparing potential intake levels with safety margins established by regulatory agencies. The results indicated that, in some cases of high consumption of herbal infusions or concentrated products, pulegone intake may approach levels of toxicological concern, particularly in vulnerable populations (contribution 2). This work emphasizes the importance of establishing more precise regulatory limits and improving labeling practices for products containing essential oils. Such studies are essential to guarantee food safety and protect consumer health while preserving the traditional use of aromatic plants.

Beyond ingredient characterization and toxicological assessment, food processing technologies also play a central role in ensuring food quality and microbiological safety. Traditionally, food preservation has relied heavily on thermal treatments. However, excessive heat may degrade vitamins, bioactive compounds, and sensory characteristics. Consequently, emerging non-thermal technologies are attracting considerable scientific and industrial interest. This Special Issue explores advancements in non-thermal processing technologies such as high hydrostatic pressure (HPP), irradiation, pulsed electric fields (PEF), ultrasound, and pulsed light. These innovative techniques allow the inactivation of pathogenic microorganisms and degradative enzymes without the application of high temperatures, thereby preserving nutritional and organoleptic quality more effectively than conventional thermal methods. Particular attention is given to the application of these technologies in infant and baby foods, a highly sensitive category due to the vulnerability of the infant immune system. The reviewed studies demonstrate that non-thermal technologies hold great potential for transforming the production of infant foods by simultaneously improving safety, preserving nutrients, and maintaining desirable sensory properties (contribution 3). Nevertheless, despite their promising potential, substantial challenges remain before large-scale industrial implementation can be achieved. High production costs, the need for regulatory validation, scalability limitations, and long-term stability studies continue to represent important barriers. In addition, consumer acceptance of foods processed using novel technologies must be carefully evaluated. Future research should therefore focus not only on technological optimization but also on economic feasibility and public perception.

Another major theme addressed in this Special Issue is the growing importance of identifying biomarkers related to oxidative stress, inflammation, and cellular metabolism in order to develop more effective nutritional recommendations and functional foods aimed at preventing chronic diseases. Understanding the relationship between dietary compounds and biological responses requires comprehensive knowledge of bioaccessibility, bioavailability, metabolism, and individual variability. Within this framework, one of the reviewed topics concerns glucosinolates and isothiocyanates, bioactive compounds mainly present in cruciferous vegetables such as broccoli, cabbage, cauliflower, and mustard. These compounds have attracted considerable interest due to their antioxidant, anti-inflammatory, and chemopreventive properties. The studies discussed in this Special Issue demonstrate that the health effects of glucosinolates and isothiocyanates depend strongly on multiple factors affecting their bioavailability. Cooking methods, intestinal microbiota composition, individual genetics, and food matrix characteristics all influence the conversion of glucosinolates into biologically active isothiocyanates. In particular, excessive thermal processing may significantly reduce myrosinase activity, thereby decreasing the formation of beneficial compounds (contribution 4). These findings highlight the complexity of translating promising in vitro and in vivo results into effective dietary recommendations. The interaction between food composition, processing conditions, and host physiology represents one of the greatest challenges in nutritional sciences and personalized nutrition.

Research into new sources of bioactive compounds with potential health benefits also represents a central topic in this Special Issue. In this regard, bergamot (*Citrus bergamia*) has been investigated as a promising source of polyphenolic compounds in the context of osteosarcopenic obesity, a condition affecting older adults characterized by the coexistence of obesity, muscle loss, and bone deterioration. Bergamot extracts rich in phenolic compounds have been associated with beneficial effects on lipid and glucose metabolism, suggesting a possible role in the management of metabolic disorders associated with aging (contribution 5). However, despite encouraging results, important limitations remain in the available clinical evidence. Additional studies are necessary to clarify compound bioavailability, establish optimal doses, and evaluate long-term efficacy within nutritional and therapeutic strategies.

Similarly, betalains have emerged as another group of promising yet underexplored bioactive compounds. Responsible for the characteristic red–violet and yellow coloration of foods such as beetroot and certain cactus fruits, betalains have attracted scientific attention due to their antioxidant, anti-inflammatory, antitumoral, and hepatoprotective properties (contribution 6). These compounds act by neutralizing reactive oxygen species and modulating signaling pathways involved in oxidative stress and inflammation. Potential beneficial effects on cardiovascular protection and metabolic regulation have also been described. Nevertheless, although preclinical studies show highly promising results, clinical evidence in humans remains limited. The need for additional investigations to determine effective doses, long-term safety, and therapeutic applications is therefore evident. Betalains represent an excellent example of the growing interest in nutraceutical compounds capable of supporting the development of functional foods and chronic disease prevention strategies.

In addition to cultivated plant sources, wild plant species are increasingly being explored as sustainable and functional resources for the pharmaceutical, nutraceutical, and food industries. One example discussed in this Special Issue is *Pistacia terebinthus*, commonly known as terebinth or cornicabra. This species is particularly rich in bioactive compounds such as polyphenols, terpenes, and fatty acids. Research highlighted in this issue demonstrates the potential application of terebinth extracts as natural antimicrobial and antioxidant agents (contribution 7). The exploitation of underutilized wild species as functional ingredients represents an innovative research line that aligns with sustainability principles and biodiversity preservation. However, technological and toxicological investigations remain necessary to confirm efficacy and safety, optimize extraction methods, and establish appropriate industrial applications. As consumer demand for natural ingredients increases, research into wild and traditional plant species may provide valuable alternatives to synthetic additives.

Once the mechanisms through which bioactive compounds exert antioxidant, anti-inflammatory, and anticancer properties have been characterized in vitro and in vivo, the next challenge is to conduct nutritional studies capable of establishing associations between dietary intake and chronic disease prevention. In this context, extra virgin olive oil has been extensively studied as a fundamental component of the Mediterranean diet. Evidence summarized in this Special Issue indicates that olive oil consumption is associated with reduced cardiovascular risk, improvements in lipid profiles, and anti-inflammatory effects (contribution 8). These beneficial properties are mainly attributed to the presence of monounsaturated fatty acids and phenolic compounds with antioxidant activity. Nevertheless, the heterogeneity of study populations, differences in dietary assessment methodologies, and variability in olive oil composition complicate the interpretation of available evidence. Consequently, further rigorous and standardized studies are required to confirm causal associations and establish more precise dietary recommendations within healthy eating patterns. This reflects a broader challenge in nutritional epidemiology: translating population-based associations into individualized and evidence-based nutritional guidance.

Beyond bioactive compounds, dietary fiber also represents a key component in the nutritional profile of foods due to its relationship with the prevention and management of colorectal cancer and intestinal diseases. Accordingly, one of the most powerful research lines currently emerging concerns the valorization of food industry by-products as sources of dietary fiber and functional ingredients. Food processing industries generate large quantities of peels, skins, pomace, seeds, and other by-products that are often discarded despite being rich in dietary fiber and bioactive compounds. The recovery and transformation of these materials into functional ingredients contributes simultaneously to sustainability, waste reduction, and the circular economy. The studies included in this Special Issue describe different physical, chemical, enzymatic, and biotechnological treatments used to extract and improve dietary fiber properties. These processes can enhance water retention capacity, texture, fermentability, and technological stability, thereby increasing nutritional and functional value (contribution 9). Furthermore, emerging technologies such as ultrasound, microwave-assisted extraction, and high-pressure processing are facilitating more efficient and sustainable recovery processes. Optimizing these technologies and developing innovative industrial applications remain important scientific challenges with substantial economic and environmental implications.

This Special Issue also addresses the importance of integrating nutritional sciences with clinical and patient-centered approaches. In this sense, celiac disease represents a particularly relevant example. Although the primary treatment for celiac disease remains lifelong adherence to a strict gluten-free diet, current research demonstrates that disease management extends far beyond dietary restriction alone. Advances in diagnostic methodologies, biomarker identification, microbiota research, and the development of complementary therapies are expanding the understanding of this autoimmune disorder (contribution 10). Potential future therapeutic approaches, such as gluten-degrading enzymes, immunomodulatory treatments, and vaccine-based strategies, are currently under investigation. In addition, psychological support, nutritional education, and improved food labeling practices are recognized as essential factors for improving patient adherence and quality of life. This multidimensional approach reflects the broader evolution of nutritional sciences toward personalized and integrative healthcare models. Nutritional interventions should not only aim to prevent disease but also to improve overall well-being and quality of life across different population groups.

Indeed, nutritional strategies are increasingly being developed not only for chronic disease prevention but also for the management of temporary physiological conditions that significantly affect daily life. One example discussed in this Special Issue concerns nausea and vomiting during pregnancy. This common condition may be alleviated through relatively simple nutritional modifications, including increased meal frequency, avoidance of prolonged fasting, prioritization of low-fat foods and complex carbohydrates, and consumption of cold liquids (contribution 11). Such strategies can help maintain adequate energy and micronutrient intake while improving maternal comfort. Importantly, these interventions demonstrate how personalized nutritional guidance can serve as an effective and safe tool to improve quality of life. However, further studies are still needed to standardize dietary recommendations and evaluate long-term effectiveness.

A recurring concept throughout the studies included in this Special Issue is the importance of interindividual variability in nutrition and food sciences. Variability between subjects has been observed in sensory perception studies involving spices and aromatic herbs, in bioaccessibility and bioavailability studies of glucosinolates and isothiocyanates, in investigations on betalain metabolism, in studies evaluating olive oil health effects, in comprehensive approaches to celiac disease, and even in nutritional management strategies during pregnancy. Factors such as digestion, food matrix interactions, microbiota composition, enzymatic activity, immune responses, and genetic variability significantly influence individual responses to dietary interventions. Consequently, one of the greatest challenges facing researchers is the high variability in scientific outcomes generated by differences among individuals.

This growing recognition of biological individuality strongly supports the development of personalized nutrition approaches. Future nutritional recommendations will likely require integration of genetics, microbiome analysis, metabolomics, lifestyle factors, and food processing effects in order to optimize health outcomes.

Overall, the contributions included in this Special Issue highlight the dynamic and multidisciplinary evolution of food science and nutrition. The growing interconnection between food systems, nutrition, health, sustainability, and technological innovation is becoming essential for the development of resilient food systems capable of supporting long-term population well-being and environmental sustainability. International collaborative research initiatives will play a crucial role in facilitating access to healthy and sustainable diets, reducing malnutrition, and promoting more efficient and equitable food systems worldwide. Focusing on topics including sensory and emotional responses to aromatic compounds, the sustainable extraction of bioactive ingredients, non-thermal food processing technologies, and personalized nutritional interventions, the studies presented herein collectively demonstrate the importance of integrative and translational research approaches.

The future of food and nutrition sciences will largely depend on the capacity to integrate technological innovation, sustainability, food safety, consumer perception, and individualized health strategies. In this context, interdisciplinary collaboration among nutritionists, food technologists, chemists, microbiologists, clinicians, toxicologists, and sensory scientists will be fundamental for addressing current and future challenges in the food sector.

Furthermore, this Special Issue emphasizes the need for more robust clinical studies, standardized methodologies, and long-term investigations capable of translating experimental evidence into practical applications for the food industry, healthcare systems, and consumers. The combination of emerging technologies, functional ingredients, and personalized nutrition approaches may ultimately contribute to healthier populations and more sustainable food systems.

We hope that the articles included in this Special Issue encourage further discussion and inspire future research focused on innovation in food science, nutrition, sustainability, and human health.

## References

[1] Delgado A., García F.P., Andrade J.M., Berry E.M., Cardoso B.R., Cash S.B., Cifuentes A., Collado M.C., Le Coutre J., German J.B. (2026). Goals in Nutrition Science 2025–2030. Front. Nutr..

